# The success of pre-hospital tracheal intubation by different pre-hospital providers: a systematic literature review and meta-analysis

**DOI:** 10.1186/s13054-017-1603-7

**Published:** 2017-02-14

**Authors:** K. Crewdson, D. J. Lockey, J. Røislien, H. M. Lossius, M. Rehn

**Affiliations:** 1 London Air Ambulance, Royal London Hospital, Whitechapel Road, London, E1 1BB UK; 2 North Bristol NHS Trust, Southmead Way, Bristol, BS10 5NB UK; 3 The Norwegian Air Ambulance Foundation, Holterveien 24, N-1441 Drøbak, Norway; 4 Department of Health Studies, University of Stavanger, Kjell Arholmsgate 41, N-4036 Stavanger, Norway

**Keywords:** Airway management, Intubation, Pre-hospital emergency care, Systemic literature review

## Abstract

**Background:**

Pre-hospital basic airway interventions can be ineffective at providing adequate oxygenation and ventilation in some severely ill or injured patients, and advanced airway interventions are then required. Controversy exists regarding the level of provider required to perform successful pre-hospital intubation. A previous meta-analysis reported pre-hospital intubation success rates of 0.849 for non-physicians versus 0.991 for physicians. The evidence base on the topic has expanded significantly in the last 10 years. This study systematically reviewed recent literature and presents comprehensive data on intubation success rates.

**Methods:**

A systematic search of MEDLINE and EMBASE was performed using PRISMA methodology to identify articles on pre-hospital tracheal intubation published between 2006 and 2016. Overall success rates were estimated using random effects meta-analysis. The relationship between intubation success rate and provider type was assessed in weighted linear regression analysis.

**Results:**

Of the 1838 identified studies, 38 met the study inclusion criteria. Intubation was performed by non-physicians in half of the studies and by physicians in the other half. The crude median (range) reported overall success rate was 0.969 (0.616–1.000). In random effects meta-analysis, the estimated overall intubation success rate was 0.953 (0.938–0.965). The crude median (range) reported intubation success rates for non-physicians were 0.917 (0.616–1.000) and, for physicians, were 0.988 (0.781–1.000) (*p* = 0.003).

**Discussion:**

The reported overall success rate of pre-hospital intubation has improved, yet there is still a significant difference between non-physician and physician providers. The finding that less-experienced personnel perform less well is not unexpected, but since there is considerable evidence that poorly performed intubation carries a significant risk of morbidity and mortality careful consideration should be given to the training and experience required to deliver this intervention safely.

## Background

There is a small but identifiable group of severely ill or injured patients in whom basic airway interventions do not provide adequate oxygenation and ventilation prior to hospital arrival [1]. To address these problems, pre-hospital advanced airway interventions, with or without the use of drugs, are frequently carried out. In the majority of cases, drugs are administered before intubation is attempted (drug-assisted intubation). If the patient is in cardiac arrest intubation may be attempted without drugs. Where drugs are used and clinicians with the appropriate skill level are available on the scene, an anaesthetic technique consisting of an induction agent and muscle relaxant, with or without the use of an opioid, is usually administered prior to intubation. In other circumstances, a sedative may be administered, with or without a muscle relaxant. It is well recognised that emergency intubation is associated with significant risk in the in-hospital setting [2–4]. Intubation performed outside the hospital is associated with a variety of complications including hypoxia, hypotension, tracheal tube misplacement, oesophageal intubation, vomiting and aspiration, cardiac arrhythmia, bleeding, and dental damage [1]. Given the complexity of pre-hospital advanced airway management, it is essential that all factors influencing intubation success are optimised prior to any intubation attempt. Advanced airway management must be performed by experienced and competent clinicians. If the appropriate skill mix is unavailable on the scene, then the most suitable alternative is likely to be basic airway interventions performed with meticulous care (with or without the use of supraglottic devices) and transfer to hospital for definitive airway management [5]. Rapid, uncomplicated, and accurate placement of the tracheal tube is one quality indicator of good advanced airway management. Monitoring the success rate of intubation is a factor describing the ability of a system to deliver high-quality airway management.

The delivery of pre-hospital advanced airway management by non-physicians remains controversial. A previous meta-analysis of pre-hospital emergency intubation published in 2012 reported a significant difference in success rates between different provider types and levels of training, with higher intubation success rates reported for physicians compared with non-physicians, and for drug-assisted intubation [6]. Some of these findings are in contradiction to those reported in another meta-analysis published in 2010 [7]. Since the publication of these two studies, both including data up to 2009, several large studies on airway management have been published, markedly increasing the number of relevant reported interventions in a relatively short time period. Data from previous meta-analyses may also be less relevant since reported data may reflect outdated practice from many years ago.

There is considerable variability in the provision of pre-hospital providers worldwide. Senior physicians commonly provide advanced pre-hospital care in many European countries whereas, outside Europe, most pre-hospital care of critically unwell patients is provided by non-physicians. The importance of safe pre-hospital advanced airway management by different providers and changes in Emergency Medical Service (EMS) provision should be informed by accurate and up-to-date data. This meta-analysis was carried out to achieve this aim.

The primary aim of this study was to systematically review the recent literature and provide updated accurate data on intubation success rates.

## Methods

### Identification and selection of studies

**Fig. 1:**
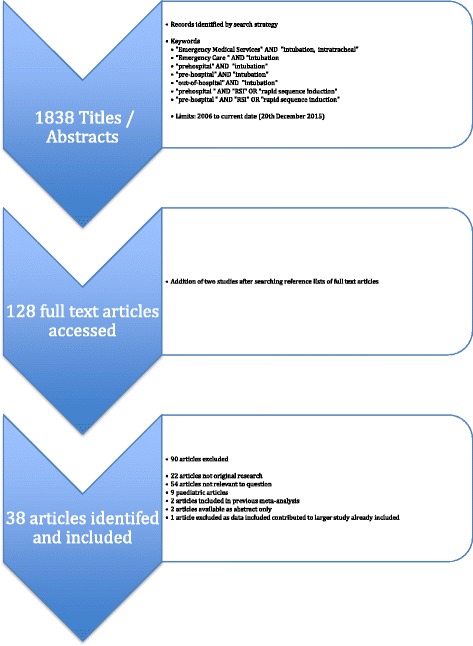
Search strategy

A systematic search of MEDLINE and EMBASE was performed using PRISMA methodology (Preferred Reporting Items for Systematic Reviews and Meta-Analyses) [8]. The search criteria are described in Table 1. All English language articles related to pre-hospital tracheal intubation published between 2006 and 2016 were identified and reviewed. Studies that reported intubation success rates as the primary outcome were included. The titles and abstracts identified by the initial search strategy were reviewed by one author (KC) to establish eligibility for inclusion in the study. A second author (MR) independently reviewed the selected studies to confirm eligibility for inclusion. The reference lists of included studies were hand searched to identify other studies meeting inclusion criteria. The full search strategy is shown in Fig. 1. Studies of paediatric tracheal intubation (described as paediatric in the title or abstract), those comparing tracheal intubation to other airway devices, and those focusing on surgical airways were excluded. Other exclusion criteria included those studies not published in English, letters to the editor, comments, editorials, and case reports. The current study has been registered with the International Prospective Register of Systematic Reviews (PROSPERO) database (registration number: CRD42015027968).

**Table 1:** Search criteria used to identify relevant studies

Keywords	
MEDLINE "Emergency Medical Services" AND "intubation, intratracheal"	
EMBASE "Emergency Care " AND "intubation/or respiratory tract intubation	
Title/abstract	
"prehospital" AND "intubation"	
"pre-hospital" AND "intubation"	
"out-of-hospital" AND "intubation"	
"prehospital " AND "RSI" OR "rapid sequence induction"	
"pre-hospital " AND "RSI" OR "rapid sequence induction"

### Data extraction and quality appraisal

The methodological quality of included studies was assessed using a validated system of internal and external criteria [9]. The data were extracted from all included studies and recorded in a standard Excel spreadsheet (2008, Microsoft Corporation). The following data were included in the study: overall intubation success rates; level of provider; number of intubation attempts and success rates by patient category (cardiac arrest, trauma, non-trauma).

### Statistical analysis

Intubation success rates are reported as median (range) unless stated otherwise. This is partly to allow for comparison with earlier studies. Individual success rates from each study are further presented in a forest plot, and the overall success rate estimated using a random effects meta-analysis for proportions. The random effects meta-analysis was used to overcome heterogeneity as it takes into account that the true effect could vary from study to study; different studies will differ in the mixture of participants and in the implementation of the intervention, and the effect sizes underlying the different studies may thus be different. The authors considered that a random effects model would be superior to a fixed effect meta-analysis, which assumes that there is one true effect size underlying all studies in the analysis. Further tests for heterogeneity were also performed, using both the *I* ^2^ and τ^2^ statistics. To assess the relationships between the intubation success rate and provider type, weighted univariate linear regression analyses were performed with intubation success rate as the dependent variable, and provider type as a dichotomous independent variable, using weights from the random effects meta-analysis. Comparison of rapid sequence induction (RSI) and non-RSI intubation success rates were performed using a Mann–Whitney *U* test and random effects meta-analysis. Results from the statistical analyses are presented as mean estimates with 95% confidence intervals (CIs). All tests were two-tailed, and statistical significance was set at a *p* value <0.05. The data were analysed using R 3.1. Meta-analysis was performed using package ‘meta in R’ [10].

## Results

The search strategy identified 1838 articles after application of the search limits described in Fig. 1. From these identified articles, the full text versions of 128 studies were accessed; 38 studies were included in the final analysis including two studies identified through searching the reference lists of other studies [11–26, 30, 35, 41–60]. Twenty-one of the studies were retrospective in methodology [12, 13, 15–18, 20, 22, 23, 25, 26, 30, 41, 43–45, 47, 52–54, 58] and 16 were prospective [14, 19, 21, 24, 35, 42, 46, 48, 49, 50, 51, 55, 56, 57, 59, 60]. All studies applied an observational study design.

**Fig. 2:**
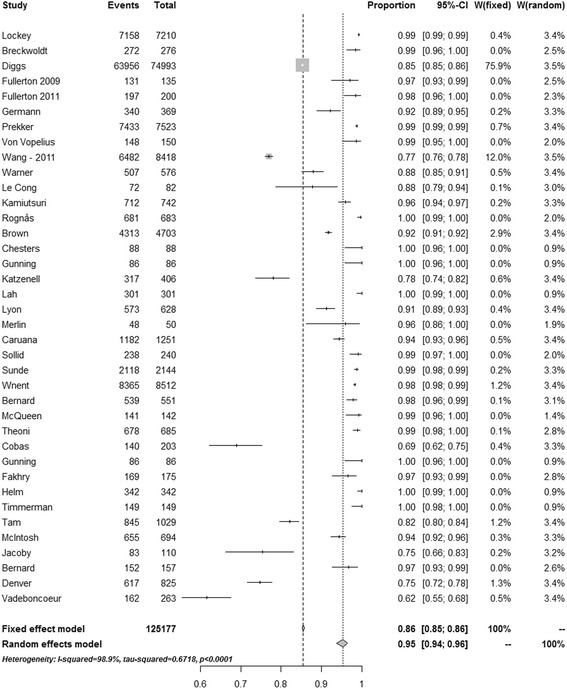
Success rates from all studies. *CI* confidence interval [11–26, 30, 35, 41–60]

**Fig. 3:**
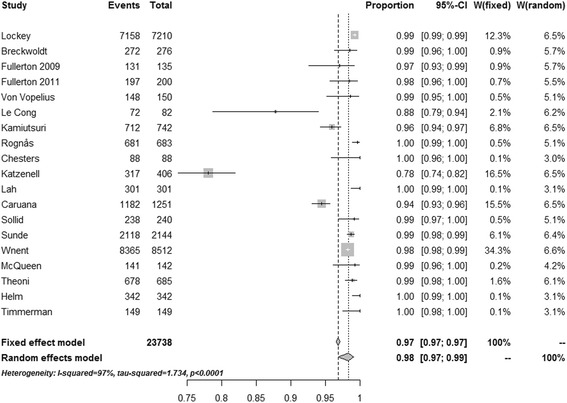
Success rates from studies describing intubation by physicians. *CI* confidence interval [12, 17, 20, 22, 30, 35, 41, 42, 43, 45–49, 51–53, 56, 57]

**Fig. 4:**
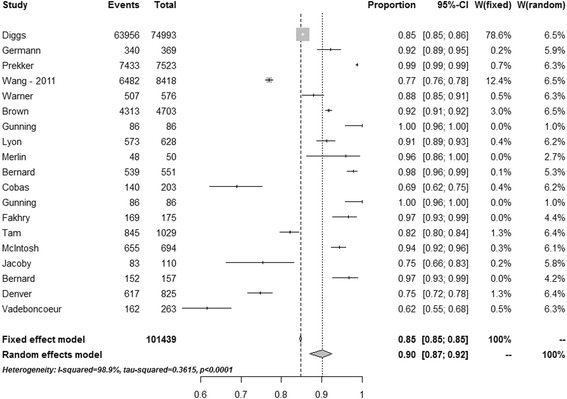
Success rates from studies describing intubation by non-physicians. *CI* confidence interval [42, 52, 53, 48, 11, 13–16, 18, 19, 21, 23–26, 44, 50, 54, 55, 58–60]

Of the 38 studies included, 19 (50%) were studies of non-physician-led services (paramedic-led or paramedic/nurse-led) and 19 (50%) were studies of services staffed by physicians. In total, 125,177 attempts at tracheal intubation were reported, which included 23,738 intubation attempts by physicians and 101,439 intubation attempts by non-physicians. The crude median (range) reported overall success rate in the studies was 0.969 (0.615–1.000). In random effects meta-analysis (Fig. 2) the estimated overall intubation success rate was 0.953 (0.938–0.965), and tests for heterogeneity showed that a fixed effects model was unsuitable for this analysis (Fig. 2). The crude median (range) reported intubation success rates for non-physicians were 0.917 (0.616–1.000) and for physicians were 0.988 (0.781–1.000) (*p* = 0.003). In random effects meta-analyses these success rates were estimated to be 0.901 (0.871, 0.925) for non-physicians and 0.984 (0.969–0.992) for physicians (Figs. 3 and 4). In weighted linear regression analysis, physician-led systems were associated with an increased success rate of 0.097 (0.035–0.159) (*p* = 0.003).

### Success rates for specific patient groups

Table 2 shows whether studies reported data from systems using an RSI drug protocol, non-RSI/‘no drug’ protocol, or data for out-of-hospital cardiac arrest. Some studies reported all three categories. Of the 38 studies included in the meta-analysis, 31 studies (15 non-physician-manned and 16 physician-manned) reported the use of an RSI drug protocol, including use of muscle relaxants. The studies reporting the use of an RSI drug protocol had an overall median (range) success rate of 0.980 (0.616–1.000). Twelve studies report data from systems using a non-RSI or ‘no drug’ protocol and/or cardiac arrest data [11–21, 24]. The median (range) success rate was 0.871 (0.639–0.989) (*p* = 0.003). Random effects meta-analysis also showed a statistically significant difference when comparing the intubation success rate for non-RSI versus RSI protocols (0.88 (95% CI 0.83 to 0.92) versus 0.96 (0.95 to 0.98); *p* = 0.00009).

**Table 2:** Category of data reported by each study

	RSI	Non-RSI	Cardiac arrest
Lockey	✓	×	×
Breckwoldt	✓	×	×
Diggs	✓	✓	✓
Fullerton 2009	×	×	✓
Fullerton 2011	✓	×	×
Germann	✓	×	×
Prekker	✓	✓	✓
Von Vopelius	✓	×	×
Wang	✓	✓	✓
Warner	✓	×	✓
Le Cong	✓	×	×
Kamiutsuri	✓	×	×
Rognås	✓	×	×
Brown	✓	×	×
Chesters	✓	×	×
Gunning 2013	✓	×	×
Katzenell	×	✓	×
Lah	✓	×	×
Lyon	×	×	✓
Merlin	✓	×	×
Caruana	✓	×	×
Sollid	✓	×	×
Sunde	✓	×	×
Wnent	×	×	✓
Bernard 2015	✓	×	×
McQueen	✓	×	×
Theoni	✓	×	×
Cobas	✓	✓	×
Gunning 2009	✓	×	×
Fakhry	✓	×	×
Helm	✓	×	×
Timmermann	✓	×	×
Tam	×	✓	×
McIntosh	✓	×	×
Jacoby	×	×	✓
Bernard 2010	✓	×	×
Denver	×	×	✓
Vadeboncoeur	✓	×	×

*RSI* rapid sequence induction [11–26, 30, 35, 41–60]

The median intubation success rate for physicians performing RSI was 0.99 (0.937–1.000) and 0.937 (0.616–1.000) for non-physicians (*p* = 0.008). Random effect meta-analysis demonstrated a success rate for physicians of 0.99 (0.98–1.0) and 0.92 (0.90–0.95) for non-physicians (*p* < 0.0001).

Nine of the 38 studies (24%) reported a median overall intubation success rate for cardiac arrest patients of 0.899 (0.748–0.988) [11–17, 19, 21]. Seven of the nine studies reported non-physician intubation for cardiac arrest patients; the median intubation success rate for these studies was 0.871 (0.78–0.988) [11, 13–16, 18, 19]. The two remaining studies for physician-led intubation had an intubation success rate of 0.983 and 0.980 [12, 17].

Eight studies reported intubation success rates for trauma patients, and the median overall intubation success rate was 0.895 (0.689–0.968) [13–15, 20, 23–26]. Seven of these studies included non-physician intubation, with a median success rate of 0.901 (0.826–0.968) [13–15, 23–26]. One study reporting data on both physician and non-physician intubation reported a success rate of 0.780 [20].

### First-pass intubation success rate

Fourteen studies reported the number of intubation attempts in addition to the overall intubation success rate. These studies included 19,178 intubation attempts; 14,913 intubations were successful at the first attempt (77.8%). For 18,630 intubation attempts, the level of provider was recorded; two studies reported mixed success rates for physicians and non-physicians [20, 22]. The median first pass success rate for intubations was 0.872 (0.776–0.9795) for physicians and 0.696 (0.634–0.973) for non-physicians.

### Level of intubator skill

The skill mix of intubators in each study was reviewed. Studies were classified as having an expert (experienced consultant anaesthetists), intermediate (physicians in training in emergency medicine and anaesthesia with some anaesthetic experience), or basic (non-physicians or those physicians with only limited anaesthetic experience) skill mix depending on the background and experience of personnel carrying out the intervention. Those studies with expert intubators reported a median intubation success rate of 0.994 (0.990–1.000). Studies including personnel with an intermediate skill mix had a median success rate of 0.986 (0.878–1.000). The reported median success rate for studies including personnel with a basic skill mix was 0.917 (0.780–1.000).

## Discussion

Pre-hospital advanced airway management remains a controversial topic, with studies providing evidence both supporting and questioning the value of this intervention. Despite ongoing debate, establishment of an early definitive airway using tracheal intubation in the prehospital setting in patients with specific indications, and those in whom the airway cannot be managed by other methods, is the currently accepted course and recommended in several national guidelines [27–29]. Strong emphasis is placed on the fact that only providers with the appropriate training and skill should undertake this intervention in the pre-hospital environment, given the number of well-recognised associated complications.

### Intubation success rates

The reporting of data for pre-hospital advanced airway management has improved significantly since the publication of previous meta-analyses in 2010 [7] and 2012 [6]. The current systematic literature review identified 38 studies published in the last 10 years (2006–2016), which included 125,177 intubation attempts for meta-analysis, more than double the number included in previous analyses. The estimated overall intubation success rate of 0.969 (0.616–1.000) in the present meta-analysis is a significant improvement when compared to 0.927 (0.882–0.961) reported by Lossius et al. [6] and 0.892 (0.877–0.905) reported by Hubble et al. [7]. This improvement was also observed in intubation success rates for non-physicians which increased from a median of 0.849 (0.491–0.990) [6] and 0.863 (0.826–0.894) [7] to 0.917 (0.616–1.000). The median overall intubation success rate for physicians in the present meta-analysis was 0.988 (0.781–1.000), showing more consistency with that reported by Lossius et al. [6] (0.991 (0.973–1.000)) rather than with the findings of Hubble et al. [7] (0.918 (0.850–0.956)). The physician data reported by the latter study represented less than 1% of the total pooled data, and included only 127 intubations [7]. This is markedly different to this meta-analysis and Lossius et al. [6], where intubation attempts by physicians account for 19.0% and 16.5% of the intubation attempts respectively. The tendency towards improvement in intubation success rates is likely to be multifactorial. The development of this subspecialty, implementation of national [27–29] and local guidelines, and formalisation of training programmes may have improved the practice of pre-hospital emergency medicine and may also have contributed to improved intubation success rates. Recent studies do suggest a standardisation of process in conjunction with increased intubation success [5, 30, 31].

### First-pass intubation success rates

Analysis of the raw data demonstrated that a first-pass intubation was successful in 77.8% of intubation attempts. The median first-pass success rate for intubations was 0.872 (0.776–0.979) for physicians and 0.696 (0.634–0.973) for non-physicians. A high first-pass success rate is associated with better outcomes in the hospital setting, and similar benefits would be expected in pre-hospital intubation. Mort [32] reports a significant increase in airway complications with more than two attempts at laryngoscopy. The incidence of hypoxaemia (defined as arterial oxygen saturation (SaO_2_) <90% or >5% decrease from baseline) changed from 11.8% with less than two intubation attempts, to 70% if there were more than two attempts at laryngoscopy [32]. The increasing use of apnoeic oxygenation both in hospital and pre-hospital reflects the recognition of this problem. Extracting robust and valid conclusions from this dataset regarding the relationship between the number of intubation attempts and outcome are impeded by the fact that few studies document how many intubation attempts were made before the intubation attempt was declared a failure or alternative airway management techniques were used.

### Intubation success rates for specific patient groups

The results from this meta-analysis are in line with the conclusions of the previous smaller dataset [6]: where drugs are used to facilitate intubation, non-physicians have a higher rate of failed intubation when compared to physicians in pre-hospital care. This may have significant safety implications since failed intubation in patients rendered apnoeic with muscle relaxants leads to risk of severe morbidity or death [24, 33, 34]. The intubation success rates for the specific patient groups of cardiac arrest and trauma are very similar in this meta-analysis, at 0.899 and 0.890, respectively. Several studies report comparable or worse intubation success rates for patients in cardiac arrest [13, 15, 16]. An exception to this is a meta-analysis by Hubble et al. [7] who demonstrated significantly higher intubation success rates in cardiac arrest patients of 91.2% versus 70.4% in non-arrest patients. A large recent study reported a doubling of the odds of intubation failure where no drugs were used [35]. It is previously documented that survival in patients who can be intubated without drugs is very poor [36].

Few studies specifically addressed intubation success rates for different patient groups. The median overall intubation success rate for cardiac arrest patients was 0.899 (0.748–0.988); 0.871 (0.78–0.988) for non-physicians and 0.981 for physicians. The median intubation success rate for trauma patients was very similar at 0.889 (0.689–0.968); the majority of these studies reported non-physician intubation. One study reporting data for both physician and non-physician intubation reported a success rate of 0.780, with a low first-pass success rate of 45%. This study was a retrospective database review of data from the Israeli Defence Forces. Patients were attended by a pre-hospital advanced life support team which was reported to be staffed by at least one military paramedic or physician [20]. The finding of comparable success rates for both trauma and cardiac arrest patients is in contrast to the previous study by Hubble et al., who reported lower intubation success rates in trauma patients compared with cardiac arrest patients [7].

### Data reporting

Despite the increase in the number of studies reporting pre-hospital advanced airway management, the data remain heterogeneous and difficult to interpret, with little standardisation between individual pre-hospital systems and practices. The studies are predominantly retrospective database studies from individual pre-hospital services or EMS registries [13, 15, 16]. A consensus-based template was developed and published in 2009 by an expert panel of pre-hospital clinicians with significant experience in advanced airway management [37]. The aim of the template was to provide a standardised method for documenting and reporting the growing data on the subject. None of the studies included in this meta-analysis reported all the variables. As the meta-analysis was designed to review the intubation success rates for different groups of pre-hospital care providers, all studies did report the highest level of provider skill on scene and the majority reported drugs used to facilitate airway management, intubation success rates, and devices used in successful airway management. Few studies described the type of ventilation used or reported on the use of end-tidal carbon dioxide. A recent focus on the mandatory use of end-tidal carbon dioxide monitoring for all intubated patients was supported by a large UK-based audit project [33], and it is included in guidelines for the provision of pre-hospital anaesthesia [5, 27, 28, 38].

### Level of intubator skill

This meta-analysis also examined the skill mix of intubators described in each study. The data demonstrated that those personnel considered to be expert intubators, i.e. experienced consultant anaesthetists, have the highest intubation success rates 0.994 (0.990–1.000) when compared with those personnel considered to have intermediate (0.986 (0.878–1.000)) or basic ability (0.917 (0.780–1.000)) (‘physicians in training in emergency medicine and anaesthesia with some anaesthetic experience’, or ‘non-physicians or those physicians with only limited anaesthetic experience’, respectively). This finding is not unexpected and is supported by Breckwoldt et al. [39] who demonstrated a significantly higher incidence of difficult intubation amongst personnel who would be considered ‘proficient’ intubators, performing a median of 18 intubations annually, compared with ‘expert’ intubators who performed a median of 304 intubations each year (*p* < 0.05). Achieving the necessary skills and maintaining currency in a pre-hospital environment can be challenging for any procedure, and tracheal intubation is a particularly good example of this challenge. It is unclear from current data how many intubations should be performed prior to being considered competent to perform this procedure in the pre-hospital setting and then subsequently, on an annual basis, to maintain currency. One study reported that healthcare personnel needed to perform a minimum of 57 intubations before achieving a 90% success rate with this procedure. Despite this, 18% of participants still required assistance after 80 intubations [40]. The authors of this meta-analysis believe that practitioners who intend to perform pre-hospital advanced airway management are unlikely to achieve high levels of competence without a period of in-hospital anaesthetic training followed by an adequate number of intubations to maintain skill levels. If personnel on scene are not competent in the provision of advanced airway intervention, careful attention should be given to optimising basic airway manoeuvres, with supraglottic airway devices used where appropriate.

### Limitations

The studies reporting pre-hospital emergency intubations are significantly heterogeneous in terms of provider and patient populations; many studies do not separate data into patient groups including cardiac arrest, non-cardiac arrest, trauma, or medical. They also often have the disadvantages of retrospective airway or trauma registry methodology. The authors acknowledge that successful intubation is only one quality indicator of advanced airway care and that other factors which have not been described in this meta-analysis may effect outcome.

## Conclusions

The overall success rate of intubation performed in the pre-hospital setting has improved, but this meta-analysis of the recent literature demonstrates a significant difference between physician and non-physician providers with or without the use of drugs. The finding that less experienced personnel perform less well is not unexpected, but since there is considerable evidence that poorly performed intubation carries a significant morbidity and mortality, careful consideration should be given to the level of training and experience required to deliver this pre-hospital intervention safely. A robust governance system is emphasised in all pre-hospital anaesthesia guidelines, and improvement and standardisation of reporting will allow better understanding of the success, process, and complications of advanced airway management.

## Abbreviations

CI: Confidence interval
EMS: Emergency Medical Service
RSI: Rapid Sequence induction
